# Fertility Preservation Strategies in Female Cancer Patients: Current Approaches and Future Directions

**DOI:** 10.3390/medicina61101794

**Published:** 2025-10-04

**Authors:** Nicolae Gică, Ioana Vișoiu, Ioana-Catalina Mocanu, Ancuța Năstac, Romina Marina Sima, Anca Maria Panaitescu, Claudia Mehedințu

**Affiliations:** 1 Faculty of Medicine, Carol Davila University of Medicine and Pharmacy, 020021 Bucharest, Romania; 2 Department of Obstetrics and Gynecology, Filantropia Clinical Hospital, 011171 Bucharest, Romania

**Keywords:** fertility preservation, trachelectomy, cryopreservation, GnRH analogs, cancer, reproductive outcomes, oncological safety

## Abstract

Fertility-sparing treatments (FSTs) have gained importance for young female cancer patients, especially those with early-stage cervical, ovarian, and endometrial cancers. However, concerns about the long-term safety of these procedures, particularly in more advanced cancers, persist. A literature review was conducted using databases such as PubMed, Scopus, and Web of Science. The search terms included “fertility preservation” and “gynaecological cancer”. Articles published between 2014 and 2024 were considered, with 39 articles cited in the paper. The inclusion criteria were female patients undergoing FST. Studies were excluded if prior treatments impacted fertility or if oncological outcomes were inadequately reported. Radical trachelectomy, laparoscopic fertility-sparing surgeries, and cryopreservation techniques, such as ovarian tissue vitrification and oocyte cryopreservation, offer viable options for preserving fertility in early-stage gynecological cancer patients. Radical trachelectomy and cryopreservation showed positive reproductive outcomes, with pregnancy rates of 30–50% in early-stage cases. GnRH analogs during chemotherapy also demonstrated benefits in maintaining fertility. Despite these advances, recurrence in more advanced stages (FIGO IA2 and beyond) remains a concern. Minimally invasive surgeries like robotic-assisted procedures demonstrated comparable fertility outcomes to traditional methods but with fewer complications. FST is a promising option for women with early-stage cancer, offering favorable reproductive and survival outcomes. However, further research is needed to confirm long-term oncological safety in advanced stages. Multidisciplinary approaches and individualized treatment planning are essential for optimizing outcomes.

## 1. Introduction

Cancer incidence among women aged 15 to 39 remains a major global health challenge, with a reported rate of 52.3 cases per 100,000 in 2019. Among this population, breast cancer and cervical cancer are the most frequently diagnosed malignancies, representing 12.7% and 8.9% of all new cases, respectively. While advances in oncologic therapies have significantly improved survival outcomes, they also carry profound implications for reproductive health. Treatments such as chemotherapy and radiotherapy can severely impair ovarian function, leading to delayed or arrested puberty, subfertility, infertility, and premature ovarian insufficiency. Understanding these risks is fundamental for providing appropriate fertility preservation (FP) counseling and interventions for young women facing cancer diagnoses [1,2].

Beyond survival, fertility plays a central role in women’s health and quality of life. The prospect of losing the ability to conceive can compound the psychological and emotional burden of a cancer diagnosis. Oncological therapies frequently compromise reproductive potential, making infertility one of the most distressing long-term sequelae. For many women, preserving the option of biological motherhood is as important as the primary cancer treatment itself, underscoring the need to integrate fertility preservation into standard oncologic care [3]. Over the past decade, a variety of FP strategies have been developed to mitigate these risks and expand reproductive choices for cancer patients. These include surgical, pharmacological, and cryopreservation techniques, each offering distinct advantages and limitations depending on cancer type, stage, and treatment plan. The use of FP has grown substantially among young patients, reflecting both increased awareness and improvements in technique. Importantly, evidence indicates that women undergoing FP procedures report higher quality of life and reduced psychological distress, highlighting the broader benefits of these interventions beyond reproduction [4,5].

To maximize outcomes, medical specialists should discuss FP options with patients before initiating cancer treatment. Adequate counseling requires both clinical knowledge and sensitivity to patients’ individual values and preferences. Developing structured guidelines and referral pathways to oncofertility specialists can ensure that patients receive timely, evidence-based, and personalized information. Such tools would enable clinicians to identify when FP is indicated, balance oncologic and reproductive priorities, and provide the emotional support needed to navigate these complex decisions [4].

This review aims to critically analyze the current strategies for fertility preservation in female cancer patients, with a particular focus on both early- and advanced-stage malignancies. In addition, it highlights existing gaps in the literature and proposes future directions for clinical practice and research. Throughout this manuscript, we use the term ***FP*** to refer to all medical, surgical, and assisted reproductive technologies intended to protect reproductive potential before or during oncological treatment. Due to heterogeneity in study design, populations, and follow-up duration, direct comparisons between FP techniques in terms of pregnancy rates, recurrence risk, and cost-effectiveness were not feasible. As such, each method is presented descriptively rather than comparatively. The terms *fertility-sparing treatment/strategies* (***FST/FSS***) refer specifically to oncological interventions, primarily surgical, that aim to preserve reproductive organs and function while maintaining cancer control. Oncological risk stratification (Low-Risk vs. High-Risk) is based on histopathological features, tumor grade, FIGO stage, and lymphovascular space invasion (LVSI), as defined by existing clinical guidelines. **FIGO (International Federation of Gynecology and Obstetrics)** staging is used as a standard system for classifying the extent of gynecologic cancers, particularly cervical, ovarian, and endometrial cancers.

## 2. Review Methodology

This paper is a descriptive, narrative review that navigates through the last decade’s FP innovations in cancer patients. The medical literature search was conducted using the PubMed scientific databases. The research was conducted using the following search formula: ((*fertility preservation*) *OR* (*preservation of fertility*) *OR* (*fertility sparing*)) *AND* ((*cancer*) *OR* (*carcinoma*) *OR* (*malignancy*) *OR* (*neoplasm*)) *AND* ((*ovarian*) *OR* (*ovary*) *OR* (*uterine*) *OR* (*uterus*) *OR* (*cervical*) *OR* (*cervix*) *OR* (*endometrium*) *OR* (*endometrial*) *OR* (*myometrium*) *OR* (*myometrial*)). Subsequently, minor adjustments were made to ensure an adequate number of results. We have selected 39 relevant studies for our review, dating from 2014 to 2024. Only full-text original articles written in the English language were chosen. We aimed to find primary studies on the topic of FP or FST in patients suffering from gynecological neoplasms (ovarian, uterine, cervical, endometrial, and myometrial) and nongynecological cancers (such as the breast, hematologic, and gastrointestinal). Inclusion criteria were female patients, human studies, FP-related interventions, and the exclusion criteria were the following: animal studies, insufficient oncological data. The research was performed by the reviewers working independently at all stages of the process. Miscommunication or conflict in the selection stage was avoided using a shared Google Sheets database. We categorized the studies based on the type of malignancy (gynecological or others) and the means of treatment or FP method employed.

## 3. Overview of Fertility Preservation Strategies in Cancer Patients

Gynecological cancers are often associated with a negative impact on female fertility. Various strategies have been employed for FP, including ovarian stimulation followed by cryopreservation of embryos or oocytes, ovarian suppression using gonadotropin-releasing hormone (GnRH) analogs, and ovarian tissue cryopreservation and transplantation (Table 1).

International societies such as the European Society for Gynecological Oncology (ESGO), European Society for Medical Oncology (ESMO), and American Society for Clinical Oncology (ASCO) have developed comprehensive guidelines outlining eligibility criteria, treatment modalities, and follow-up protocols for FP in gynecologic oncology. These recommendations were used as a benchmark for evaluating the appropriateness of fertility-sparing approaches described in the literature. Table 2 below summarizes the core criteria and treatment strategies recommended for gynecologic malignancies.

## 4. Critical Appraisal of Fertility Preservation Strategies

### 4.1. Gynecological Malignancies and FP

#### 4.1.1. Hormonal Treatment

To begin with, a study on 8 patients analyzed the effects of using GnRH analogs during chemotherapy. In 2 cases, cryopreservation of ovarian tissue and oocytes was performed at the onset of treatment, and these patients later experienced spontaneous pregnancies. Four out of eight patients treated with GnRH analogs also achieved spontaneous pregnancies. While these findings suggest a potential protective effect of GnRH analogs, the small sample size considerably limits the generalizability of the results. Moreover, the coexistence of cryopreservation in some patients makes it difficult to isolate the independent contribution of GnRH analogs to fertility preservation. The proposed mechanism, an initial gonadotropin surge followed by pituitary desensitization and the creation of a hypogonadotropic, prepubertal-like environment, remains biologically plausible. However, its clinical relevance is still debated, to establish if this suppression truly reduces ovarian susceptibility to chemotherapy-induced damage [6].

Alternative hormonal therapy for FP involves the use of progestins. Although endometrial cancer (EC) and its precursors are more common in postmenopausal women, they can also occur in younger patients who may wish to maintain fertility, making standard surgical management unsuitable. One study compared oral megestrol acetate (MA) alone versus MA combined with a levonorgestrel-releasing intrauterine system (LNG-IUS) in patients with early-stage EC. Both groups achieved high complete remission rates, with subsequent pregnancies reported in over half of the patients who attempted conception. However, the combination therapy did not significantly improve fertility outcomes compared to MA alone and was associated with a higher risk of vaginal hemorrhage. These findings suggest that MA remains an effective fertility-preserving option, while the additional benefit of LNG-IUS is questionable. Nonetheless, the relatively small sample size and limited follow-up period caution against overgeneralization, and further studies are needed to clarify whether LNG-IUS offers long-term advantages in selected patients [7].

However, both studies discussed involved small cohorts and lacked randomization. The retrospective nature and limited follow-up reduce the strength of the conclusions regarding oncological safety and fertility outcomes.

#### 4.1.2. Controlled Ovarian Hyperstimulation

Emergency FP is increasingly used when time is limited before initiating cancer therapy. Conventional controlled ovarian stimulation (COS) requires 2–5 weeks, often delaying treatment by up to 5 weeks if started in the early follicular phase. To overcome this, “random-start” COS protocols have been introduced, allowing stimulation at any point in the menstrual cycle. Retrospective studies in cancer patients preparing for gonadotoxic therapy demonstrated the feasibility of this approach, with successful oocyte retrieval and good-quality embryo production within approximately 2 weeks. Protocols often combined GnRH antagonist regimens with recombinant FSH, and in estrogen-sensitive tumors, letrozole was added to minimize estrogen exposure. Reported fertilization rates were high, and embryo quality was not compromised compared with conventional IVF. However, current evidence is limited to small retrospective cohorts with short follow-up and heterogeneous cancer types, lacking robust control groups. While promising for urgent FP, the true safety, long-term reproductive outcomes, and oncological implications of random-start COS remain insufficiently studied, underscoring the need for prospective trials before broad implementation [8].

#### 4.1.3. Ovarian Sparing Surgery

A 25-year UK cohort study of 88 pediatric patients reported that 38 underwent ovarian-sparing surgery, with tumor enucleation followed by ovarian reconstruction. The study demonstrated excellent overall survival (97%) in pediatric ovarian neoplasms, though adverse outcomes were linked to aggressive histologies such as ovarian carcinoma and therapy-resistant desmoplastic small round cell tumors. These results underscore the potential of ovarian-sparing approaches in preserving both fertility and hormonal function. However, while the survival rates are reassuring, the findings largely reflect outcomes in patients with benign or less aggressive disease, limiting their applicability to high-risk cases. Moreover, the study highlights the importance of long-term surveillance, as even benign disease requires ongoing monitoring to safeguard reproductive health. Future directions, such as integrating artificial intelligence to refine risk stratification and management pathways, may enhance personalized care, though these remain speculative and require validation in larger multicenter studies [9].

Ovarian germ cell tumors (OGCTs) represent 20–25% of ovarian neoplasms, with malignant subtypes (MOGCTs) accounting for about 5%. For patients of reproductive age, FP is often feasible through unilateral oophorectomy combined with omentectomy. While omentectomy serves an important role in staging and reducing metastatic risk, its preventive use remains controversial, as the procedure may carry additional surgical morbidity without always providing clear benefits in low-stage disease. Consequently, the balance between oncological safety and FP must be carefully individualized, emphasizing the need for tailored surgical decision-making supported by long-term outcome data [25].

Unilateral oophorectomy provides the benefit of preserving fertility and hormonal function by retaining one ovary, thereby minimizing disruption to endocrine balance while addressing the primary pathology. However, this approach is not without limitations. The risks of residual disease, recurrence, or the development of a new tumor in the preserved ovary remain significant considerations. Thus, careful patient selection is essential, with fertility-sparing surgery best reserved for those with favorable tumor characteristics and adequate oncological safety margins. Long-term surveillance is equally important to ensure that the balance between oncologic control and FP is maintained [6].

Fertility-sparing surgery (FSS) has been associated with high conception rates and a low incidence of premature ovarian failure, making it an attractive option for young patients. Nevertheless, its potential risks must be carefully discussed during pre-treatment counseling, particularly given the possibility of residual disease or recurrence. While advanced-stage malignant MOGCTs represent only 20–30% of cases, the evidence for conservative management in this subgroup remains limited. Although FSS is increasingly regarded as a safe and feasible approach, its oncologic reliability is not yet fully substantiated, underscoring the need for larger prospective studies to validate long-term outcomes [10].

Most of the available evidence comes from single-center, retrospective studies. While outcomes are encouraging, the lack of prospective validation and small sample sizes limits the strength of recommendations for widespread use.

#### 4.1.4. Uterine Procedures

Preserving the uterus in oncological patients is crucial because it allows for the possibility of future pregnancies after cancer treatment, which can be an important factor in long-term quality of life after remission.

##### Endometrial Curettage

A 2020 study evaluated a fertility-sparing strategy in women with atypical hyperplasia/endometrial intraepithelial neoplasia (AH/EIN) and early-stage endometrial cancer (EC), combining hysteroscopic resection and curettage with subsequent progestin therapy (megestrol acetate). The protocol showed encouraging outcomes, with approximately half of the patients who attempted conception achieving pregnancy, some spontaneously and others via assisted reproductive techniques. Live birth rates, however, remained modest (around 30–38%), and adverse outcomes such as miscarriage or intrauterine demise were also reported. These findings highlight the potential of combining conservative surgery with hormonal therapy, but the evidence is based on a small cohort and limited follow-up. Consequently, while the approach appears feasible, its oncologic safety and long-term reproductive benefits require validation in larger prospective trials [11].

##### Myomectomy

An 11-year study of 27 patients with uterine smooth muscle tumors of uncertain malignant potential (STUMP) who underwent myomectomy reported seven subsequent pregnancies. These findings suggest that fertility-sparing approaches may be feasible for women wishing to preserve reproductive potential. However, oncological outcomes in this setting remain unclear, as current evidence is limited and lacks long-term follow-up. The rarity of STUMP and the heterogeneity of its biological behavior further complicate the development of clear guidelines, highlighting the need for multicenter studies to better define both reproductive and oncologic safety [12].

##### Uterine Preservation

Low-grade serous ovarian carcinoma (LGSOC) is a rare subtype of epithelial ovarian cancer, accounting for 2.5% of all ovarian cancers and 5% of serous ovarian carcinomas. Given that many patients are of reproductive age, FP (FP) may be a relevant consideration. For early-stage disease (FIGO IA–IC1), fertility-sparing surgery—typically unilateral salpingo-oophorectomy with omentectomy and lymphadenectomy while preserving the uterus—is regarded as an option. Uterine preservation is particularly important, as it allows for both spontaneous conception and assisted reproduction, including oocyte donation or embryo transfer. However, while pregnancies have been reported following FSS, the oncological safety of this approach remains uncertain. Data on recurrence risk and long-term survival after conservative management are limited, underscoring the need for cautious patient selection and further research [13].

In a cohort of women with early-stage EOC 36 underwent FSS while 51 received radical surgery. FSS, defined as preservation of the uterus and contralateral ovary, allowed for future natural conception. Among the FSS group, 17 natural pregnancies were reported in 15 patients (93.7%), underscoring the reproductive potential of this approach. Nevertheless, while these findings are encouraging, the small sample size and retrospective design limit the strength of the conclusions. Radical surgery continues to represent the gold standard for EOC, and although FSS appears safe in carefully selected women with stage IA disease, its role in more advanced stages remains uncertain. Rigorous follow-up is therefore essential when offering conservative management [14].

A retrospective study evaluated uterine preservation combined with bilateral salpingo-oophorectomy in six patients with early-stage infiltrative mucinous ovarian cancer (IA–IC3). Two patients developed recurrence involving the pelvic peritoneum and uterine serosa, with one death reported, raising significant concerns about oncologic safety. Although two women achieved pregnancies via oocyte donation, the small cohort and high recurrence rates highlight the risks associated with uterine preservation in this aggressive histological subtype. These findings suggest that, while fertility may be achievable in selected cases, the approach remains experimental and should be pursued with extreme caution. Larger studies are needed to clarify whether any subgroup of patients with infiltrative mucinous tumors could safely benefit from uterine preservation [15].

For uterine adenosarcoma, total hysterectomy remains the standard surgical treatment. However, uterine preservation has been explored as a fertility-sparing option in younger patients, particularly given the generally favorable prognosis of low-grade disease. A retrospective study of 31 women with stage I uterine adenosarcoma reported outcomes in seven patients who underwent conservative procedures, such as hysteroscopic excision or dilatation and curettage. Among these, three remained disease-free, two had persistent intrauterine disease, and two experienced recurrence. One patient successfully conceived and delivered. Importantly, recurrence was strongly associated with sarcomatous overgrowth, which emerged as a significant risk factor (OR 13.3, *p* = 0.027). These findings suggest that FP may be cautiously considered in carefully selected patients, but sarcomatous overgrowth should be viewed as a contraindication to uterine preservation due to its high recurrence risk [16].

Data on uterine preservation in malignant settings remain scarce and should be interpreted with caution.

#### 4.1.5. Cervical Procedures

Cervical cancer is the fourth most common cancer among women globally, with 85% of cases occurring in developing countries. Radical hysterectomy with pelvic lymphadenectomy is the standard of care for patients with early-stage disease (FIGO 2009 IA with lymphovascular space invasion IB1) who do not wish to preserve their fertility. Trachelectomy is a surgical procedure to remove the cervix. There are two types of trachelectomy: simple trachelectomy and radical trachelectomy. In a simple trachelectomy, only the cervix is removed, while in a radical trachelectomy, the cervix, surrounding tissue, and the upper portion of the vagina are also removed. The 2018 NCCN guidelines recommend performing radical trachelectomy and pelvic lymphadenectomy, with or without sentinel lymph node mapping, in patients who wish to preserve their fertility. Several publications have documented the safety and feasibility of both vaginal and laparotomic approaches to radical trachelectomy. As minimally invasive surgery has gained popularity, numerous investigators have reported on the safety and feasibility of minimally invasive radical trachelectomy.

The comparison of survival outcomes between open and minimally invasive radical trachelectomy remains an important area of investigation. Evidence from a prospective randomized trial demonstrated inferior oncologic outcomes with minimally invasive radical hysterectomy compared to open surgery in early cervical cancer. Similarly, a national registry study reported higher 4-year mortality with minimally invasive surgery (9.1%) than with open surgery (5.3%) (HR = 1.65; 95% CI 1.22–2.22; *p* = 0.002), with a median follow-up of 45 months. Notably, the adoption of minimally invasive techniques after 2006 coincided with a yearly 0.8% decline in 4-year relative survival (95% CI 0.3–1.4%; *p* = 0.01). These findings raise serious concerns about the oncologic safety of minimally invasive radical hysterectomy. By extension, while minimally invasive radical trachelectomy has been hypothesized to offer outcomes comparable to the open approach, robust data are lacking. Until high-quality evidence becomes available, the assumption of equivalent oncologic safety for minimally invasive trachelectomy should be approached with caution [26,27].

A 2019 study analyzed 359 women with early-stage cervical cancer who underwent fertility-sparing trachelectomy, of whom 121 (33.7%) pursued fertility treatments postoperatively. The findings underscored the prognostic significance of tumor characteristics: patients with small tumors (≤2 cm), without nodal metastasis, deep stromal invasion, or high-risk histology achieved favorable outcomes, with a 5-year recurrence rate of 2.8%. Conversely, women with adverse tumor features had a substantially higher recurrence risk (16.6% at 5 years). Importantly, postoperative treatments did not significantly affect pregnancy outcomes. The study highlighted the need for a multidisciplinary infrastructure—including experienced gynecologic oncologists and pathologists capable of intraoperative frozen section assessment of nodes and margins—to ensure both oncologic safety and reproductive success. While fertility-sparing trachelectomy offers excellent outcomes in carefully selected patients, its application in higher-risk cases remains controversial and requires cautious evaluation [17].

A 2022 study assessed fertility outcomes after robot-assisted radical trachelectomy in 135 women with early-stage cervical cancer. Of the 88 patients who actively attempted conception, 80% achieved pregnancy, most through natural conception (84%), with a smaller proportion via assisted reproduction (16%). In total, 103 pregnancies and 76 live births were reported, including one twin gestation, with only a few premature deliveries before 32 weeks. These results suggest that robot-assisted radical trachelectomy can yield high pregnancy rates and favorable obstetric outcomes. However, the lack of long-term oncologic data and the absence of detailed obstetric follow-up, such as gestational age at delivery in some cases, limit the strength of these conclusions. Further prospective studies are needed to confirm both the reproductive safety and the oncologic reliability of this minimally invasive approach [18].

Loop electrosurgical excision procedure (LEEP) is a fertility-sparing option for patients with stage IA1 cervical squamous cell carcinoma (SCC). Both the 2020 NCCN guidelines and supporting literature endorse LEEP and cold knife conization (CKC) as safe and effective treatments in the absence of lymph-vascular space invasion (LVSI). LEEP offers advantages such as lower morbidity, reduced discomfort, and lower costs, but is limited by a higher risk of positive margins and interpretive challenges caused by thermal artifacts. In contrast, CKC is generally preferred in clinical practice for its superior margin assessment. Reproductive outcomes following LEEP are favorable: in one study of 119 patients, 75% of those attempting conception were successful, with 93.3% delivering at term and only one miscarriage reported due to cervical incompetence. Compared with CKC, LEEP appears more fertility-friendly, as CKC carries a greater risk of cervical insufficiency and adverse obstetric outcomes. Nonetheless, long-term surveillance remains crucial for ensuring oncologic safety after either procedure [28].

**Figure 1:**
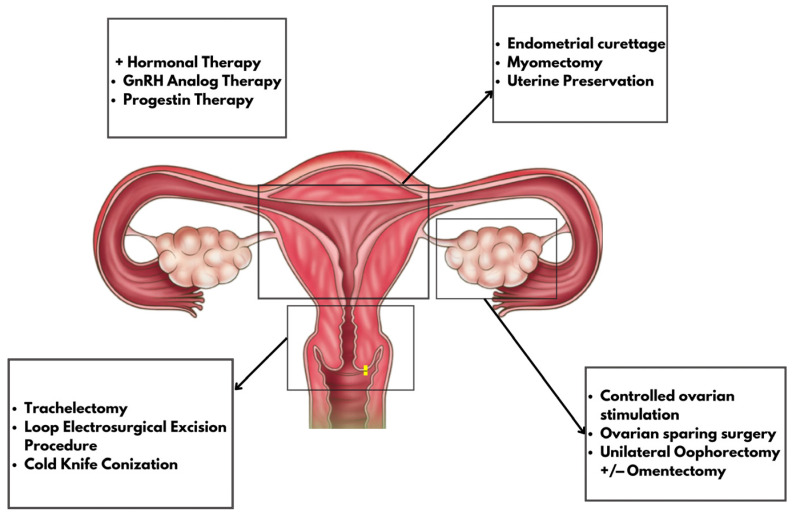
Main procedures used for FP in gynecological cancers.

Despite promising pregnancy outcomes, the oncologic safety of minimally invasive trachelectomy remains debated. Furthermore, existing studies differ in surgical technique, staging criteria, and follow-up intervals, making comparisons difficult and conclusions tentative (Figure 1).

### 4.2. Non-Gynecological Malignancies and FP

Under the umbrella term of “non-gynecological” cancers, we rounded up all malignancies except those affecting the ovary, uterus, and adnexa. One example of such cancer is breast cancer, being the most prevalent cancer in the female population, but we also took notice of hematologic, gastrointestinal, and conjunctival tissue malignancies. Suffering from such a pathology deeply affects patients’ quality of life, and also chemotherapy causes secondary amenorrhea in 20–80% of premenopausal women, depending on their age and treatment regimen (type and duration) [19].

Fertility impairment can also arise in childhood, particularly among pediatric cancer patients who develop primary ovarian insufficiency (POI) following gonadotoxic treatments. Alkylating agents, such as cyclophosphamide, and radiotherapy are the most frequent culprits. In the study by Colino et al. [29], 83% of patients had not undergone any form of oocyte or ovarian tissue preservation prior to chemotherapy or the onset of POI. Among the cohort, 53% (16 patients) reported amenorrhea, while an additional 23% experienced amenorrhea or oligomenorrhea lasting at least four months. In 87% of cases, hormone replacement therapy was the preferred fertility-preserving measure. These findings underscore both the vulnerability of pediatric patients to treatment-related infertility and the persistent underutilization of proactive FP strategies. Greater emphasis on early counseling and implementation of cryopreservation methods before gonadotoxic therapy could improve reproductive outcomes in this high-risk population [29].

#### 4.2.1. Ovarian Stimulation and Neoadjuvant Treatment Delay

In many non-gynecological malignancies, the preferred fertility-sparing protocol is that of ovarian hyperstimulation in order to produce oocytes for freezing before starting chemoradiotherapy.

A key concern in FP for breast cancer patients is whether COS delays initiation of therapy and impacts prognosis. A study of 82 women with stage II–III breast cancer compared those who underwent ovarian stimulation (STIM) before neoadjuvant therapy (NAT) with a control group who declined fertility preservation. Patients in the STIM group were generally younger, nulliparous, and had less advanced disease, reflecting a degree of selection bias. Importantly, the time to treatment initiation did not differ significantly between groups: NAT began after an average of 39.8 days in the STIM group and 40.9 days in controls, suggesting that COS does not compromise timely access to therapy. Among the 34 patients undergoing ovarian stimulation, 16 opted for oocyte retrieval, 20 for embryo cryopreservation, and 2 for both. These findings indicate that reproductive function can be preserved without delaying cancer treatment, although the influence of patient characteristics and tumor biology on outcomes should be interpreted cautiously [19].

#### 4.2.2. Use of GnRH Analogs During Chemotherapy

One of the methods used to trigger ovulation in FP is the administration of GnRH analogs. In the context of hematologic malignancies, particularly lymphoma, gonadal function may already be impaired before oncologic treatment, raising questions about the feasibility of FP. A study stratified lymphoma patients into groups with favorable and unfavorable disease parameters. Following ovarian stimulation with recombinant FSH, hCG triggering, and oocyte maturation using the GnRH antagonist buserelin, patients with favorable parameters yielded a higher median number of mature oocytes (11 vs. 9, *p* = 0.01) despite requiring lower FSH doses (*p* = 0.04). These results suggest that disease biology can influence FP outcomes, with patients in more favorable categories responding better to stimulation. While FP should be offered broadly, tailoring strategies to individual disease characteristics is essential to maximize reproductive potential while ensuring that oncologic priorities remain uncompromised [20].

Goldrat et al. (2019) [21] investigated the impact of letrozole-associated controlled ovarian hyperstimulation (Let-COH) in breast cancer patients compared to infertile women. The study assessed oocyte quality through hormonal levels and the expression of genes such as *HAS2*, *PTGS2*, and *GREM1*. While an estrogen-rich environment is generally favorable for oocyte maturation, it raises concerns in hormone-dependent cancers due to the potential risk of tumor stimulation. In the Let-COH group, ovulation triggered with GnRH agonists yielded significantly higher hormonal levels compared to hCG triggering (median 194.5 vs. 64.4 ng/mL, *p* < 0.001). This effect, however, was not observed in the control group of infertile women. These findings suggest that Let-COH combined with GnRHa triggering may optimize oocyte quality in breast cancer patients while potentially minimizing estrogen exposure. Still, the clinical significance of these molecular markers and their correlation with long-term fertility and oncologic outcomes remain uncertain, underscoring the need for further validation [21].

Although GnRHa has traditionally been used in ovarian stimulation protocols, its administration during chemotherapy has been explored as an additional fertility-preserving strategy. The proposed mechanism involves pituitary desensitization, leading to suppression of FSH and LH secretion and thereby inducing a prepubertal-like hormonal state thought to protect ovarian follicles. However, primordial follicles lack both FSH and GnRH receptors and are not directly regulated by gonadotropins, making this mechanism biologically questionable. Moreover, if GnRHa truly reduced the cytotoxic effects of chemotherapy on the ovaries, one would expect a parallel reduction in the overall efficacy of chemotherapy, which has not been observed in clinical outcomes. These discrepancies highlight the ongoing controversy surrounding the protective role of GnRHa, suggesting that its benefit may derive from indirect or alternative mechanisms rather than direct inhibition of follicular recruitment [30].

This method could provide fertility protection against the gonadotoxic effect of breast cancer chemotherapy and immunotherapy, all while avoiding the delay in treatment. We believe that this might have a tremendous impact on the evolution of younger patients, who oftentimes suffer from malignancies with a higher degree of aggressivity.

#### 4.2.3. Oocyte and Embryo Cryopreservation

Oocyte and embryo cryopreservation are key FP methods, particularly for women undergoing sterilizing or gonadotoxic treatments. Oocytes are retrieved following ovarian stimulation, while embryos are obtained through IVF of harvested oocytes. Although initially established as an oncofertility tool, oocyte freezing is now more frequently performed electively by women wishing to postpone childbearing. A comparative study found that women in the oncofertility group were both younger (31.3 ± 5.8 vs. 37.0 ± 2.9 years, *p* < 0.001) and more likely to pursue oocyte freezing (62.5% vs. 19.9%, *p* < 0.001) than those in the elective group. Interestingly, cancer patients had significantly lower FSH levels (median 4 vs. 6.9, *p* < 0.001), but no differences were observed in AMH or BMI, suggesting a comparable ovarian reserve. These findings underscore that while cancer patients often present with favorable reproductive parameters due to younger age, timely intervention remains critical to optimize outcomes. At the same time, the growing use of elective cryopreservation reflects broader societal trends, raising questions about how best to balance medical necessity and personal choice in FP strategies [22].

For patients in need of urgent gonadotoxic treatment, In Vitro Maturation (IVM) after immature oocyte retrieval has no different outcome concerning the time of retrieval (early follicular, late follicular, or luteal phase). However, for estrogen-dependent cancers, this process has to happen under antiestrogenic therapy. There are concerns relating to the correlation between ovarian stimulation and estrogen-related cancer relapse [23].

#### 4.2.4. Ovarian Tissue Cryopreservation

Ovarian tissue cryopreservation (OTC) followed by transplantation (OTT) represents an increasingly used FP option, even in patients exposed to chemotherapy. Poirot et al. (2019) [24] reported ovarian function restoration in 83% of women one year post-transplant (95% CI, 67.0–94.4), with no significant difference between those who underwent OTC before, during, or after chemotherapy. Of the 31 patients included, 22 had received chemotherapy, most involving alkylating agents—either bifunctional (e.g., cyclophosphamide) or monofunctional (e.g., dacarbazine). Notably, the live birth rate was higher among women with prior chemotherapy exposure (32%; 7/22) compared with those without (0%; 0/9). Overall, 13 pregnancies occurred in 8 patients, resulting in 8 live births, 3 miscarriages, 1 biochemical pregnancy, and 1 elective abortion; 12 of these pregnancies were spontaneous, with only 1 achieved through assisted reproduction. These findings suggest that prior chemotherapy does not preclude favorable outcomes after OTC/OTT, although the paradoxically better results in pretreated patients may reflect selection bias or confounding factors. The limited cohort size and heterogeneous treatment exposures underscore the need for larger, controlled studies to clarify the impact of chemotherapy on reproductive success following OTC [24].

This procedure can also be recommended for pediatric cases, as the treatment focus has broadened from survival-only to considering the future reproductive needs of the patient. In prepubertal patients, due to the smaller size of the ovaries, oophorectomy is recommended instead of the conventional decortication [31].

In our view, cryopreservation would be the most effective fertility-sparing option for female cancer patients. Both oocyte and embryo cryopreservation have demonstrated high success rates and offer a well-established, widely available solution for preserving fertility before cancer treatment begins. Additionally, recent advancements in vitrification have significantly improved the survival rates of cryopreserved oocytes and embryos, making this approach a reliable and preferred option for many patients.

**Figure 2:**
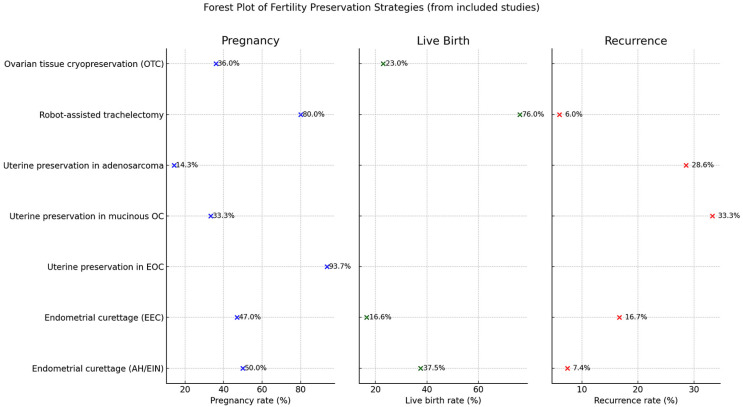
Forest plot of FP strategies.

As survival rates have risen in both pediatric and adult oncological populations, we have to consider their desire for reproduction. Many young women have a strong desire for motherhood; therefore, fulfilling their wish by helping them maintain reproductive function increases their quality of life (Figure 2).

The decision to pursue FP in female cancer patients is a delicate and complex process that requires a careful balance between the desire for future parenthood and the primary goal of an oncological cure. While FP strategies offer a crucial opportunity for a better quality of life post-treatment, their implementation must not compromise the patient’s prognosis or increase the risk of cancer recurrence. Although current evidence suggests that ovarian stimulation protocols are largely safe and do not adversely affect oncological outcomes, most data are retrospective and limited by small sample sizes and short follow-up periods, and the risk of relapse in advanced stages of disease is still unknown. Another critical aspect is the timing of FP, as any delay in initiating definitive cancer treatment could allow the disease to progress. For this reason, rapid-start stimulation protocols have been developed to minimize these delays. The long-term safety of more experimental techniques, such as ovarian tissue transplantation, remains an area of active research.

Beyond the clinical and technical aspects, the FP process is intertwined with significant ethical, psychological, and accessibility issues. Ethically, the principle of informed consent is paramount; patients must be fully aware of the potential risks and benefits, as well as the success rates, of each procedure. The psychological burden on patients is immense, as they are simultaneously confronting a cancer diagnosis and making life-altering decisions about their reproductive future. Discussions about FP can cause significant distress, highlighting the importance of providing comprehensive psychological support throughout the process. Furthermore, issues of accessibility often create barriers to care. The high cost of FP procedures and the lack of insurance coverage can make these options unavailable to many patients, exacerbating existing health disparities.

Therefore, a comprehensive discussion involving a multidisciplinary team is essential to tailor FP strategies to each patient’s specific cancer type, stage, and prognosis. This ensures that FP is a viable option that not only aligns with and does not jeopardize their overall oncological safety but also addresses the complex ethical dilemmas and supports the patient’s emotional well-being and financial situation.

### 4.3. Future Directions and Research Prospects

#### 4.3.1. Development of the Artificial Ovary

During the last decade, there were some important steps taken towards the development of an artificial ovary. Although the research is limited, the advancements are notable. Artificial ovary technology offers a promising alternative to conventional FP methods, especially for patients with prepubertal cancer or premature ovarian failure. Unlike ovarian tissue cryopreservation, which carries the risk of reintroducing malignant cells, the artificial ovary involves transplanting isolated follicles with or without a biological scaffold. This method has shown success in restoring endocrine function, follicular development and achieving pregnancies in animal and human trials. However, improvements in follicular recovery rates, scaffold design, transplantation techniques, and genetic safety are needed for consistent human application. Additionally, advancements in understanding the ovary’s mechanical and biochemical properties, folliculogenesis, and imaging techniques will further enhance its clinical potential [32,33].

#### 4.3.2. Robotic Surgery Advantages

In the era of robotic surgery advancements, FSS are highly influenced, too. Robotic surgery might increase the chance of preserving fertility by enabling a more precise dissection and selective preservation of pelvic structures during exenterative procedures. In this context, the partial conservation of the distal vagina and the urogenital diaphragm has been associated with the maintenance of sexual function and a reduced risk of empty pelvis syndrome. Furthermore, in selected cases, type I and IIA posterior exenterations may allow the creation of a colorectal anastomosis, thus avoiding permanent colostomy. Reconstructive strategies for both urinary and digestive continuity are tailored to the extent of disease and the patient’s prior treatments, particularly in those with a history of radiotherapy. Although pelvic exenteration remains a highly radical approach, the integration of robotic-assisted techniques and individualized reconstructive options can provide significant functional benefits beyond oncologic control [34]. Similarly, minimally invasive pelvic exenteration has been reported as a safe and feasible alternative to open surgery, offering lower morbidity, shorter hospital stays, and faster recovery while maintaining comparable oncologic outcomes. Evidence indicates that anterior exenteration is associated with better prognosis than total exenteration, and that both minor and major postoperative complications significantly affect survival and quality of life. Importantly, the use of omental patches and reconstructive techniques, including flaps or mesh grafts, has been emphasized to prevent empty pelvis syndrome and to mitigate the mutilating nature of the surgery. Regarding urinary reconstruction, incontinent diversions such as ileal conduits or ureterostomies remain the standard due to their lower complication rates, whereas continent reconstructions, although functionally superior and potentially beneficial for younger patients, are associated with higher risks of fistula formation and septic complications. These findings highlight how minimally invasive and robotic approaches may not only preserve oncological safety but also support strategies aimed at functional sparing and improved postoperative quality of life [35].

The studies that we used were often limited to a smaller number of patients. Therefore, further research needs to include wider cohorts, especially when studying the newest innovative surgical treatments. Moreover, for cancers managed with fertility-sparing techniques, there is a critical need for further investigation into long-term oncological outcomes following surgery. One of the primary limitations of existing FP strategies is the lack of long-term oncological outcome data. Many studies are retrospective and include short follow-up periods (often <5 years). Cancer recurrence risk, particularly in higher-stage or hormone-sensitive tumors, remains insufficiently studied. For instance, while FSS appears feasible in early-stage endometrial or cervical cancers, concerns remain regarding occult metastasis and local recurrence, particularly in high-grade or deeply invasive cases. It was surprising to find that GnRH analogs not only help preserve ovarian function but also have a positive short-term effect on breast cancer remission, highlighting the need for further research in this area. Additionally, emerging technologies such as artificial ovaries, stem cell-derived gametes, and regenerative uterine engineering may offer new avenues for patients currently lacking safe FP options. Aligning future clinical trials with international recommendations (ESGO, ESMO, ASCO) will ensure consistency and improve the quality of evidence in fertility-sparing oncology. Future meta-analyses or large prospective studies are needed to enable direct comparison of FP methods using standardized outcome reporting.

### 4.4. Ethical and Psychological Challenges

Oncofertility poses unique ethical dilemmas. For pediatric or adolescent patients, consent and autonomy are limited, making decision-making complex and emotionally charged. Socioeconomic factors may limit access to advanced FP methods (e.g., oocyte cryopreservation or ovarian tissue banking), generating inequity in outcomes. These disparities must be acknowledged, and healthcare systems should ensure equitable access to oncofertility services. In view of the available evidence, long-term survival, recurrence, and obstetric outcomes must be carefully weighed when considering fertility-sparing strategies. This applies not only to well-established procedures such as trachelectomy, but also to more experimental approaches, including ovarian tissue transplantation and uterine preservation in rare or aggressive malignancies. While these methods offer the possibility of reproductive success, their oncologic safety remains uncertain in several contexts, underscoring the need for cautious patient selection, thorough counseling, and long-term follow-up. Recent evidence highlights that the psychosocial consequences of FP versus loss of fertility are complex and evolve over time. Cancer survivors frequently report uncertainty regarding their fertility status and reproductive options, irrespective of whether FP was undertaken. While FP can initially provide reassurance and a sense of control, it may also introduce additional concerns related to the sufficiency of cryopreserved material, the safety and effectiveness of ART, and the emotional burden of unsuccessful procedures. Conversely, lack of FP can lead to regret, grief, and heightened distress about lost reproductive opportunities. Importantly, survivors describe profound effects on romantic relationships, partner communication, and dating, with infertility often associated with feelings of loss or guilt. Thus, FP cannot fully buffer against the psychological impact of infertility, and both pathways, undertaking or not undertaking FP, are associated with distinct emotional trajectories that warrant careful counseling and ongoing psychological support [36].

## 5. Conclusions

In conclusion, FP should become an essential consideration in the comprehensive care of cancer patients, especially given the reproductive risks associated with many cancer treatments. Advances in techniques such as oocyte and embryo cryopreservation, ovarian tissue freezing, and fertility-sparing surgeries offer a range of options for preserving reproductive potential. However, timely referral to oncofertility specialists and individualized counseling are critical to ensuring patients understand their options before initiating treatment. As research continues to evolve, it is important to further discover FP strategies and also make them more accessible and effective for all women diagnosed with a malignancy. Integrating these approaches can significantly improve the quality of life for cancer survivors by guarding their fertility and, alongside that, confidence and femininity.

## Figures and Tables

**Table 1:** Summary of main articles and the methods used and analyzed.

Paper	Method	Malignancy	Sample Size	Pregnancy Rate	Live Birth Rate	Study Duration	Outcomes
Xiong et al., 2022 [6]	Unilateral vs. bilateral Oophorectomy	Gynecological	28480 patients	Not reported	Not reported	17 years	Unilateral oophorectomy associated with better survival in stage IA patients < 50 years; bilateral approach yielded worse outcomes in stage II–III
Xu et al., 2023 [7]	Megestrol Acetate(MA) vs. MA + Levonorgestrel Intrauterine System(LNG-IUS)		26 patients MA, 28 patients MA + LNG-IUS	Not significantly different between groups	Not reported	Median 31.6 months follow-up (range 3.1–94)	Complete response at 32 weeks: 57.1% (MA) vs. 61.5% (MA + LNG-IUS), with no significant difference between groups
Kim et al., 2015 [8]	Random-start Controlled Ovarian Hyperstimulation		22 cancer patients (6 in early follicular phase, 11 in late follicular phase, 5 in luteal phase) vs. 40 controls	Not reported	Not reported	Between 10 and 14 days follow-up	Random-start controlled ovarian hyperstimulation was feasible across cycle phases, though fertility outcomes were not reported
Arthur et al., 2021 [9]	Surgical management of ovarian tumors (pediatric): salpingo-oopherectomy n = 21 (21%), ovary excision n = 33 (33%), ovary sparing tumourectomy n = 34 (34%), and cyto-reductive extirpation in 2 cases (2%)		88 patients	Not reported	Not reported	1990–2018 (18 years)	Recurrence rate 10%, with overall survival of 97% over 18 years
Dellino et al., 2020 [10]	Fertility-sparing surgery for malignant ovarian germ cell tumors		28 patients	100% (5/5 patients with spontaneous pregnancies)	Not reported	90 months follow-up	One recurrence observed; ovarian function preserved in all others, with 100% spontaneous pregnancy rate in those attempting conception
Ayhan et al., 2020 [11]	Endometrial curettage in Atypical Hyperplasia(AH)/Endometrial Intraepithelial Neoplasia(EIN) and Endometrioid Endometrial Cancer(EEC)		57 patients (27 AH/EIN-group A, 30 EEC-group B)	50% in group A, 47% in group B	37.5% in group A, 16.6% in group B; in group B, 1 patient experienced intrauterine exitus	January 2007–October 2018	Recurrence rate higher in EEC (16.7%) vs. AH/EIN (7.4%)
Şahin et al., 2019 [12]	Myomectomy/Hysterectomy in smooth uterine muscle of uncertain malignant potential(STUMP)		57 patients	7 pregnancies out of 10 attempts (in patients who underwent myomectomy)	Not reported	January 2006–December 2017 Median 57 (16–125) months follow-up	7 recurrences of STUMP and 1 leiomyosarcoma transformation reported
Petiot et al., 2024 [13]	Uterine preservation in Low Grade Serous Ovarian Carcinoma		26 patients, 73% in stage FIGO III	Not reported	Not reported	January 2000–May 2022	Uterine preservation was feasible in early-stage disease, but hysterectomy remained necessary in advanced stages
Chen et al., 2020 [14]	Uterine preservation in epithelial ovarian carcinoma		87 patients, 36 undergoing fertility-sparing surgery	93.75% (15 out of 16 patients)	Not reported	January 2005–December 2014	Fertility-sparing surgery has to be considered in early-stage epithelial ovarian carcinoma
Gouy et al., 2017 [15]	Uterine preservation combined with bilateral salpingo-oophorectomyin infiltrative mucinous ovarian cancer		6 patients	33.33% (2 patients)	Not reported	1976–2016	33.3% recurrence rate, questioning oncologic safety
Lee et al., 2017 [16]	Uterine preservation in early-stage uterine adenosarcoma		31 patients, 7 undergoing uterine preservation surgery	1 patient-vaginal delivery at term (14.28%)	Not reported	1998–2014 Median 32 months follow-up	Persistent disease observed in 2 patients, while 2 patients experienced disease recurrence
Machida et al., 2020 [17]	Trachelectomy for early-stage cervical cancer		401 patients	Not reported	Not reported	2009–2013	Five-year recurrence rates were 2.8% in low-risk tumors (≤2 cm) vs. 16.6% in high-risk tumors, supporting trachelectomy mainly in low-risk cases
Ekdahl et al. 2022 [18]	Robot-assisted radical trachelectomy for early stage cervical cancer		149 patients	70 out of 88 conceived (80%)	76 livebirths in 81 pregnancies	2007–2019, Median follow-up 58 months	6% recurrence rate; NB: short postoperative cervical length was associated with impaired fertility
Chien et al., 2017 [19]	Ovarian stimulation(OS) and neoadjuvant treatment delay in stage II-II breast cancer		82 patients were included (34 undergoing OS and 48 control)	6 underwent embryo transfer, two pregnancies in one patient	2 live births	April 2010–February 2017 Median follow-up 79 months	Recurrence and death rates were similar in both groups; no adverse effect on survival compared with controls
Volodarsky-Perel et al., 2020 [20]	Ovarian stimulation in women with lymphoma	Non-Gynecological	141 patients	Not reported	Not reported	2009–2018	Lower mature oocyte yield observed in advanced-stage lymphoma and in patients with poor biochemical markers
Goldrat et al., 2019 [21]	Mature oocyte cryopreservation following letrozole associated controlled ovarian hyperstimulation (Let-COH) in breast cancer patients		47 (23 Let-OH, 24 control)	Not reported	Not reported	December 2012–February 2017	Let-COH resulted in reduced estradiol and increased testosterone in the follicular fluid relative to conventional COH. The oocyte environment was suboptimal under hCG triggering, whereas GnRHa triggering enhanced oocyte quality
Kira et al., 2022 [22]	Oocyte vitrification in breast cancer (elective vs. oncofertility preservation)		40 cancer patients vs. 327 elective	Not reported	Not reported	2009–2018	Oocyte vitrification outcomes in breast cancer patients were comparable to elective preservation controls
Creux et al., 2017 [23]	In vitro maturation(IVM) in cancer patients requiring urgent chemotherapy		165 patients	Not reported	Not reported	January 2003–December 2015	IVM was feasible at all cycle stages, offering a rapid fertility preservation option
Poirot et al., 2019 [24]	Ovarian tissue preservation in various cancer patients (especially hematological malignancies)		31 patients (22 with previous chemotherapy)	36%	23%	2005–2015	Prior chemotherapy did not influence ovarian function recovery or pregnancy incidence; the sole parameter associated with outcome variation was the quantity of ovarian tissue retrieved

**Table 2:** Overview of Fertility-Sparing Recommendations in Gynecological Malignancies. Abbreviations: LVSI—lymphovascular space invasion; SLNB—sentinel lymph node biopsy; LNG-IUS—levonorgestrel intrauterine system.

Society	Cancer Type	Eligibility Criteria	Recommended Fertility-Sparing Treatment	Follow-Up Protocol
**ESGO** (2023)	Cervical cancer	Stage IA1-IB1, tumor < 2 cm, no LVSI, negative nodes	Conization (IA1), simple/radical trachelectomy ± SLNB (IB1)	MRI every 6 months; colposcopy, cytology
**ESMO** (2022)	Endometrial cancer	Grade 1, stage IA, no myometrial invasion (MRI), no LVSI	High-dose progestins ± LNG-IUS; hysteroscopic resection	Hysteroscopy + biopsy every 3–6 months
**ASCO** (2021)	Ovarian cancer	Stage IA–IC1, low-grade, unilateral tumors, no genetic risk	Unilateral salpingo-oophorectomy ± staging	Imaging and CA-125 every 3–6 months

## Data Availability

No new data were created or analyzed in this study. Data sharing is not applicable to this article.
